# Development and chromosomal characterization of interspecific hybrids between common buckwheat (*Fagopyrum esculentum*) and a related perennial species (*F. cymosum*)

**DOI:** 10.1270/jsbbs.22063

**Published:** 2023-05-17

**Authors:** Mari Sugiyama, Miyu Norizuki, Shinji Kikuchi, Yasuo Yasui, Katsuhiro Matsui

**Affiliations:** 1 Shimane Agricultural Technology Center, 2440 Ashiwata-cho, Izumo, Shimane 693-0035, Japan; 2 Graduate School of Horticulture, Chiba University, 648 Matsudo, Matsudo-shi, Chiba 271-8510, Japan; 3 Graduate School of Agriculture, Kyoto University, Kitashirakawa Oiwake-cho, Sakyou-ku, Kyoto 606-8502, Japan; 4 Institute of Crop Science, National Agriculture and Food Research Organization (NARO), 2-1-2 Kannondai, Tsukuba, Ibaraki 305-8518, Japan; 5 Institute of Life and Environmental Sciences, University of Tsukuba, 2-1-2 Kannondai, Tsukuba, Ibaraki 305-8518, Japan

**Keywords:** embryo rescue, genomic *in situ* hybridization, interspecific hybrids, sterility

## Abstract

Common buckwheat (*Fagopyrum esculentum*) is an annual self-incompatible plant that is widely grown. The genus *Fagopyrum* comprises more than 20 species, including *F. cymosum*, a perennial that, unlike common buckwheat, is highly resistant to excess water. In this study, we developed interspecific hybrids between *F. esculentum* and *F. cymosum* via embryo rescue, to improve undesirable traits of common buckwheat, such as low tolerance to excess water. The interspecific hybrids were confirmed by genomic *in situ* hybridization (GISH). We also developed DNA markers to confirm the identity of the hybrids and if genes derived from each genome were inherited by the next generation. Observations of pollen indicated that the interspecific hybrids were essentially sterile. Unpaired chromosomes and abnormal segregation during meiosis were likely responsible for the pollen sterility of the hybrids. These findings could facilitate buckwheat breeding to produce lines that can withstand harsh environments with wild or related species in the genus *Fagopyrum*.

## Introduction

Common buckwheat (*Fagopyrum esculentum*) is an outcrossing annual crop that is widely cultivated throughout the world (Ohsawa 2020). Buckwheat grains are used to make several types of food such as noodles, pasta, and bread. The grains are rich in nutrients and bioactive compounds, including flavonoids, which are good for human health (Kreft *et al.* 2020, Matsui and Walker 2020).

However, buckwheat yield is low and unstable compared to other major crops, which is attributed to environmental complications such as water stress (Zhou *et al.* 2018). In addition, common buckwheat possesses many inferior agricultural traits, such as preharvest sprouting, allergenic proteins, and self-incompatibility. To improve these agricultural traits, many landrace and mutated populations have been used, and undesirable traits have been improved by conventional and molecular breeding (Matsui and Yasui 2020a, Ohsawa 2020). However, the use of these materials is limited because some important agricultural traits, such as resistance to excess water stress and self-compatibility, are lacking in these *F. esculentum* resources.

One way to improve these undesirable traits is to generate interspecific hybrids, although various undesirable traits could be introduced into the hybrids as well. We previously developed the self-compatible buckwheat line ‘Norin PL1’ (Matsui and Yasui 2020a) via an interspecific cross between *F. esculentum* and *F. homotropicum*; ‘Norin PL1’ shows self-compatibility and possesses long homostyle flowers. Self-compatible lines are often used for genetic analysis and to develop mutant lines (Matsui and Yasui 2020a).

The genus *Fagopyrum* (*Polygonaceae*) is composed of approximately 20 species that are classified into two major groups: cymosum and urophyllum (Ohsako and Li 2020). The cymosum group includes two cultivated buckwheat species, common buckwheat (*F. esculentum*) and Tartary buckwheat (*F. tataricum*), as well as a perennial buckwheat (*F. cymosum*). Each species possesses different useful traits that affect cultivation, nutrient levels, taste, and food processing quality. *F. cymosum* is known to be highly tolerant to high temperatures and over watering, making it an ideal material for breeding buckwheat that can be grown in converted fields in the face of anticipated global warming. Furthermore, perennial growth habit of *F. cymosum* would reduce the farmer’s labor and could also produce a perennial buckwheat plant that exhibits a permanent hybrid vigor (Zhou *et al.* 2018). Therefore, many researchers have tried to produce interspecific hybrids using this species. Chen *et al.* (2004) performed interspecific hybridization between *F. tataricum* and *F. cymosum*, and the resulting interspecific hybrids were perennial. In the current study, we developed interspecific hybrids between *F. esculentum* and *F. cymosum* using embryo rescue and validated the identity of the hybrids using genomic *in situ* hybridization (GISH) and DNA markers. We also observed the behavior of chromosomes in the hybrids during meiosis to determine whether the interspecific hybrids could be used as parental materials to improve common buckwheat.

## Materials and Methods

### Plant materials

Both *F. esculentum* and *F. cymosum* are heterostylous self-incompatible species with two different flower types, pin and thrum (Matsui and Yasui 2020b, Ohsako and Li 2020). In pin plants, the flowers have long styles and short stamens. In thrum plants, the flowers have short styles and long stamens. Both intra-morph self-incompatibility and flower morphology are controlled by a single genetic locus named the *S* locus; thrum plants are heterozygous (*Ss*) and pin plants are homozygous recessive (*ss*) at this locus. To produce interspecific hybrids, we used pin plants of Japanese landraces *F. esculentum* var. ‘Yokota-zairai’, ‘Izumo-zairai’, and ‘Matsue-zairai’ and Japanese cultivars ‘Izumonomai’ and ‘Hitachiakisoba’ as the pistil parents, and the thrum plant diploid *F. cymosum* (a landrace from Shimane prefecture) as the pollen parent.

### Embryo culture

Crossing was carried out between *F. esculentum* pin plants as the pistil parents and *F. cymosum* thrum plants as the pollen parents, both of which were grown in pots in a greenhouse. Three to five days after pollination, the flowers were collected and the petals removed. The ovaries, which enlarged to approximately 3 to 4 mm, were collected, sterilized in 70% (v/v) ethanol for 1 minute, and rinsed in sterile distilled water. The ovule was carefully harvested from the ovary under aseptic conditions and placed on solid 1/2 MS medium with the concentrations of potassium nitrate and ammonium nitrate reduced by half compared to the original MS medium (Murashige and Skoog 1962) and supplemented with 0.1% (w/v) naphthaleneacetic acid (NAA), 2% (w/v) sucrose, and 0.02% (w/v) gellan gum (pH 5.8).

The cultures were maintained at 25°C under a fluorescent light (FL40SS ENW/37 F2; Panasonic) with a 16-h photoperiod. Regenerated plants were subcultured on hormone-free 1/2 MS medium with 2% (w/v) sucrose and 0.02% (w/v) gellan gum (pH 5.8). After acclimatization, the rooted plants were transferred to pots and grown in a greenhouse.

### Chromosome preparation and GISH

To observe mitotic chromosomes, fresh roots were collected from plant cuttings of one hydroponically cultivated plant. The roots were pretreated with 0.045% 8-hydroxyquinoline at 10°C for 6 h and fixed in 3:1 (v/v) ethanol–acetic acid at room temperature for 3 days. The fixed samples were stored in 70% ethanol at 4°C until use. To observe meiotic chromosomes, fresh young flower buds (approx. 0.5–2 mm long) were collected and immediately fixed in 3:1 (v/v) ethanol–acetic acid. Mitotic/meiotic chromosomes were observed using the squash method described by Wang *et al.* (2015).

Total DNA was extracted from young leaves of *F. esculentum* and *F. cymosum* using a DNA Extraction Kit (DNAs-ici!-R, RIZO Inc., Tsukuba, Japan) and stored at –30°C until use. GISH probes were prepared from these DNA samples using Biotin-Nick Translation Mix or DIG-Nick Translation Mix (both from Sigma, USA). The *F. esculentum* probe was labeled with Dig-rhodamine, and the *F. cymosum* probe was labeled with biotin- FITC. GISH analysis of mitotic and meiotic chromosomes was performed as described by Muakrong *et al.* (2018). GISH signals were captured under an OLYMPUS BX-53 fluorescence microscope equipped with a CoolSNAP MYO CCD camera (Photometrics, USA) and processed using MetaVue/MetaMorph version 7.8 and Adobe Photoshop CS3 v10.0.1 software. At least five well-spread chromosome slides with clear GISH signals were used to determine chromosome number and composition in the interspecific hybrids.

### Development of DNA markers

Total DNA was isolated from leaves of common buckwheat, *F. cymosum* and interspecific hybrids using a DNeasy Plant Mini Kit (Qiagen). We selected several genetic loci related to the flavonoid biosynthetic pathway because *F. esculentum* and *F. cymosum* appear to possess similar flavonoid biosynthetic pathways and similar genes related to flavonoid biosynthesis, with several differences in gene structure or sequence. In common buckwheat, several genes and putative genes in the flavonoid biosynthetic pathway have been reported (e.g. Kim *et al.* 2013, Koja *et al.* 2018), such as genes encoding chalcone synthase (CHS), chalcone isomerase (CHI), flavanone 3-hydroxylase (F3H), flavonoid 3ʹ-hydroxylase (F3ʹH), flavonol synthase (FLS), dihydroflavonol 4-reductase (DFR), anthocyanidin synthase (ANS), rhamnosyl transferases (RT), anthocyanidin reductase (ANR), and leucoanthocyanidin reductase (LAR). We chose *F3H*, *DFR*, and *ANR*, as these genes were expected to be present in only a single or few copies in *F. cymosum* based on the common Buckwheat Genome DataBase (BGDB; Yasui *et al.* 2016; http://buckwheat.kazusa.or.jp).

Gene structures from common buckwheat were estimated based on sequence data in BGDB and GenBank (NCBI). The genomic sequences were obtained from BGDB, and the coding sequences were obtained from GenBank (NCBI). As for DFR, we had already identified two loci in buckwheat and identified genome sequences in each gene (Katsu *et al.* 2017), and in this research the sequence information was used. Several regions across an intron were amplified with primers designed based on information from BGDB. The amplified fragments were sequenced, and primers were designed that can amplify both regions in *F. esculentum* and *F. cymosum* (Supplemental Fig. 1)

The DNA was amplified by PCR using Ex-Taq Polymerase (Takara). Thermocycling conditions were an initial 94°C for 2 min and 35 cycles of 94°C for 30 s, 60°C for 30 s, and 72°C for 60 s. Primers used to detect polymorphisms between *F. esculentum* and *F. cymosum* are listed in Supplemental Table 1.

Sequencing was performed with an Applied Biosystems SeqStudio Genetic Analyzer (Applied Biosystems), and the data were assembled using Sequencher software (Gene Codes Corporation).

The developed makers were checked with more than four different buckwheat cultivars and eight *F. cymosum* plants including parental lines, and all 26 interspecific hybrid plants which were selected from 46 developed hybrids were checked hybridity with the developed F3H marker.

### Estimation of pollen fertility

To estimate pollen fertility, mature pollen grains were stained with acetocarmine and examined under a microscope (OLYMPUS BX43). Pollen that was stained uniformly and well was considered to be fertile, while empty pollen that was not stained or pollen that was partially and insufficiently stained was considered to be sterile. Pollen collected from a flower was analyzed on a single slide, and the analysis was repeated with pollen from at least three flowers. More than 300 pollen grains per line were scored as fertile or sterile based on staining quality.

## Results and Discussion

### Development of interspecific hybrids between *F. esculentum* and *F. cymosum*

Both *F. esculentum* and *F. cymosum* are heterostylous self-incompatible plant species with two different flower types, pin and thrum (Matsui and Yasui 2020b, Ohsako and Li 2020). To reduce the possibility of self-incompatibility, we performed crosses between different flower types. In addition, since *F. cymosum* rachises, which support the flowers and seeds, tend to be brittle, we used *F. esculentum* as the style parent and *F. cymosum* as the pollen parent (Table 1). We used five different accessions (including landraces) of common buckwheat to reduce the effect of genetic background. The landrace ‘Yokota-zairai’ showed a higher probability of producing interspecific hybrids than the other cultivars and landraces. Although it is possible that the other cultivars and landraces failed to produce interspecific hybrids because the number of plants used was small, these lines were less suitable for producing interspecific hybrids than Yokota-zairai under our culture methods (Table 1). We therefore used interspecific Yokota-zairai × *F. cymosum* hybrids for subsequent analysis.

In this research, we didn’t measure and evaluate any phenotypes of each regenerated plant. This is because it is difficult to arrange the growth stages of these plants and they sometime show different phenotypes even if they were derived from the same cultured plant.

### Confirmation of the hybrids by GISH

**Fig. 1.**
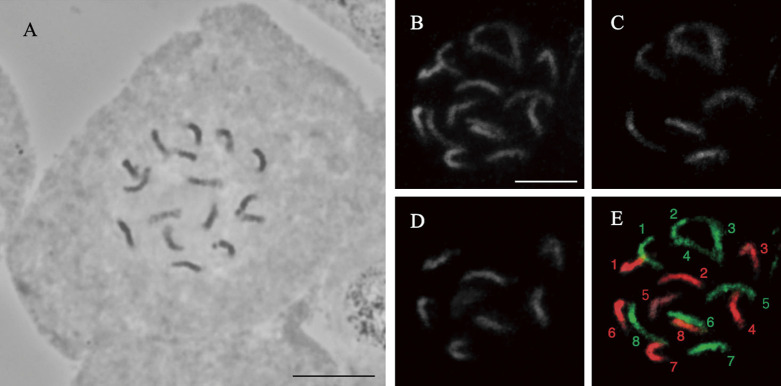
Mitotic chromosomes of an interspecific hybrid between *F. esculentum* and *F. cymosum*. (A) Sixteen chromosomes were observed in a somatic cell of the root tip, suggesting that the plant is a diploid hybrid. (B)–(E) GISH analysis. The chromosomes (B) were hybridized with the *F. cymosum* genomic probe (C) and the *F. esculentum* genomic probe (D), and the different colored chromosomes were merged (E), i.e., green chromosomes are from *F. cymosum*, and red chromosomes are from *F. esculentum*. The hybrid possessed eight chromosomes each from the two species. Note: Numbers indicate the chromosome count, not the chromosome number. Scale bars, 10 μm.

Although common buckwheat is generally self-incompatible, it sometimes produces seeds from self-pollination (Matsui and Yasui 2020b). To confirm that the plants that we produced by embryo rescue were interspecific hybrids containing both chromosomes, we performed GISH analysis. The interspecific hybrids contained 16 chromosomes in their root cells, as did *F. esculentum* and *F. cymosum* (Fig. 1A). Half of the chromosomes (eight chromosomes) appeared green following GISH, indicating that the genomic DNA probe of *F. cymosum* hybridized with *F. cymosum* chromosomes (Fig. 1B, 1C, 1E). The remaining eight chromosomes were red, indicating that the *F. esculentum* genomic DNA probe hybridized with *F. esculentum* chromosomes (Fig. 1B, 1D, 1E). Thus, the hybrid individuals bred in this study were confirmed to be diploid hybrids containing chromosomes from both *F. esculentum* and *F. cymosum*.

### Development of markers to quickly identify the hybrids

**Fig. 2.**
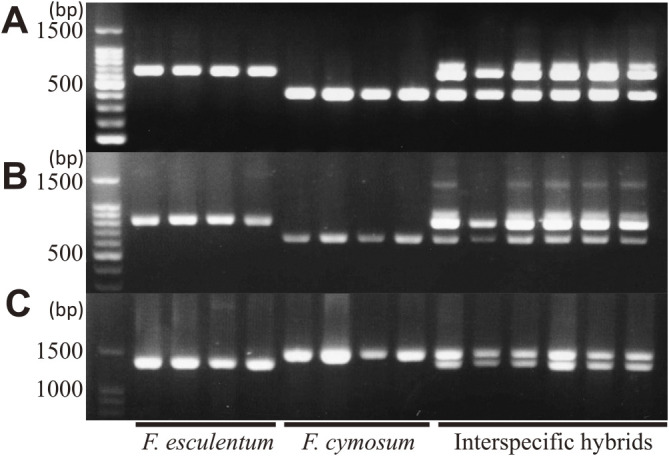
Newly developed markers show polymorphism between *F. esculentum* and *F. cymosum*. (A), *F3H*; (B), *DFR*; (C), *ANR*. The primer position of each locus is shown in Supplemental Fig. 1.

We developed DNA markers showing polymorphism between *F. esculentum* and *F. cymosum* for use as tools to quickly identify the interspecific hybrids. We designed primers across introns in each gene (*FeF3H*, *FeDFR1a*, and *FeANR*; Supplemental Fig. 1, Supplemental Table 1) because introns in genes from common buckwheat often differ in sequence and length even between plants in the same cultivar. We identified differences in the sequence length of each gene including introns. All markers showed polymorphism between *F. esculentum* and *F. cymosum*, although some extra bands or bands of different lengths appeared in the interspecific hybrids using primers for *FeF3H* and *FeDFR1a* (Fig. 2). The reason for the appearance of extra bands in hybrids is not known, but it is likely that other loci encoding similar sequences of these genes are readily amplified in interspecific hybrids. Actually, the extra band of interspecific hybrid contained similar sequence to *FeF3H* and *FcF3H* (data not shown). *F. esculentum* and *F. cymosum* are self-incompatible plants, indicating that they have many sequence differences not only in intron but also exon. By examining the polymorphism of the parental lines at the loci targeted by these markers or in their flanking regions, the hybridity and inheritance of chromosomes and genes by the progeny can be identified even when different lines are used to produce interspecific hybrids.

Using the developed F3H marker 26 regenerated plants that showed good growth were examined, and all were confirmed to be interspecific hybrids.

### Pollen fertility and chromosome behavior during mitosis in the interspecific hybrids

**Fig. 3.**
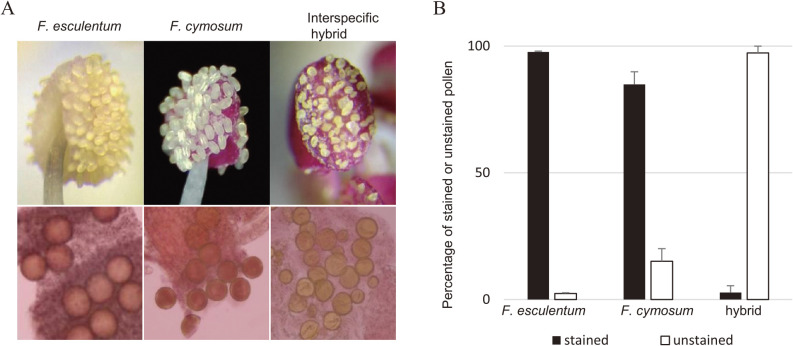
Estimation of pollen fertility of an interspecific hybrid. (A), Shape and size of pollen on the anther (top) and acetocarmine stained pollen (bottom) from *F. esculentum*, *F. cymosum*, and the interspecific hybrid. *F. esculentum* and *F. cymosum* pollen was uniform and was stained by acetocarmine, while pollen from the interspecific hybrids is variable and was not stained by acetocarmine. (B), Evaluation of the rate of fertile and sterile pollen based on acetocarmine staining.

To introduce traits of the interspecific hybrids into *F. esculentum* or *F. cymosum*, the interspecific hybrids must be crossed with plants of these species. To investigate whether an interspecific hybrid could be used as a pollen parent, we examined the pollen fertility of a plant that shows good growth by acetocarmine staining. The pollen fertility of the interspecific hybrid was significantly lower than that of *F. esculentum* and *F. cymosum* (Fig. 3A). The percentage of unstained pollen considered to be sterile increased to 97.3% (N = 494 pollen) in the hybrid compared to 2.3% in *F. esculentum* (N = 424) and 15.0% in *F. cymosum* (N = 340) (Fig. 3B).

**Fig. 4.**
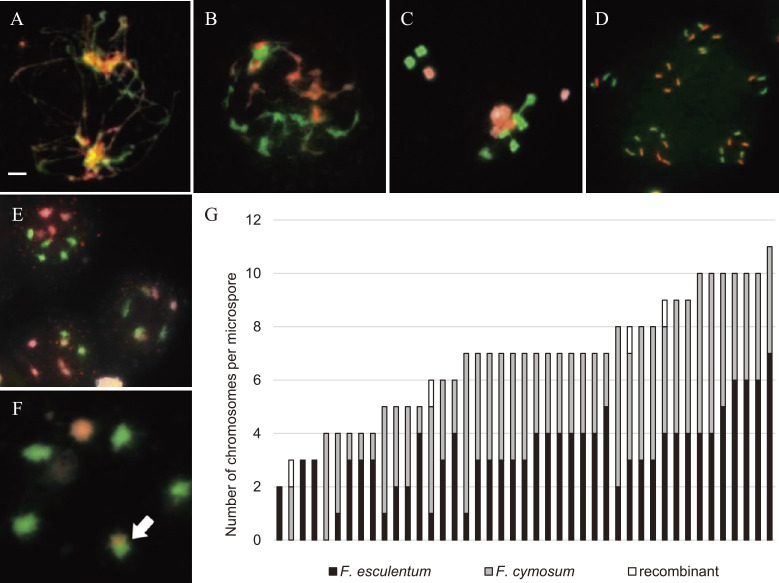
Behavior of chromosomes of a diploid hybrid during meiosis and chromosome composition in microspores. (A), Pachytene. (B), Late pachytene/diplotene. (C), Meiotic metaphase. (D), Telophase II. (E), Microspores with different chromosome numbers and compositions. (F), Microspore with a putative recombinant chromosome containing chromosome segments from both species (arrow). (G), Range of variation in chromosome number and composition in 43 microspores. Scale bar, 10 μm.

To determine whether the low fertility of the hybrid was caused by chromosome segregation abnormalities, we performed GISH analysis during meiosis and in the microspores of the interspecific hybrid lines (Fig. 4). During meiosis, definite chromosome pairing was not observed, and 16 univalent chromosomes formed (Fig. 4A–4C). At the end of meiosis, we often observed the formation of more than four daughter cell nuclei (Fig. 4D). Each microspore resulting from this type of meiosis possessed different chromosome numbers and compositions (Fig. 4E), and putative recombinant chromosomes were detected (Fig. 4F). Thus, even though chromosome pairing between the two species was not observed in this study (Fig. 4A–4C), the identification of putative recombinant chromosomes suggests that pairing occurred at low frequencies and in limited chromosomal regions. Because mature pollen normally develops following meiotic chromosome segregation, abnormal chromosome number and genetically unbalanced chromosome composition (Fig. 4G) may cause pollen sterility.

### The use of *F. cymosum* for common buckwheat breeding

Many interspecific hybrids have been successfully developed within the *Fagopyrum* genus (e.g. Asaduzzaman *et al.* 2009, Chen *et al.* 2004, 2018, Fesenko *et al.* 2022, Hirose *et al.* 1991, Matsui *et al.* 2003, Samimy *et al.* 1996, Suvorova *et al.* 1994, Ujihara *et al.* 1990, Woo *et al.* 1999a, 1999b). *F. cymosum* has many useful agricultural traits, such as a perennial habitat, excess water stress resistance and high rutin content (Zhou *et al.* 2018). Therefore, *F. cymosum* has been used as a material to produce interspecific hybrids with the cultivated species common buckwheat and Tartary buckwheat. By contrast, although interspecific hybrids between *F. esculentum* and *F. cymosum* have been produced (e.g. Fesenko *et al.* 2022, Hirose *et al.* 1991, Suvorova *et al.* 1994, Ujihara *et al.* 1990, Woo *et al.* 1999b), most of these interspecific hybrids showed low seed set and produced low fertility pollen. In the current study, we also developed interspecific hybrids between *F. esculentum* and *F. cymosum*, and almost all the hybrid lines showed low seed set and produced sterile pollen.

Our chromosome analysis during meiosis and analysis of microspores of interspecific hybrids revealed abnormal chromosome segregation, which was likely a critical factor that made this interspecific hybrid sterile. Our GISH analysis clearly revealed the presence of chromosomes of both species in the hybrid (Fig. 1B–1E), indicating that DNA sequence variation accumulated in this line. Common buckwheat and *F. cymosum* have different genome sizes (Nagano *et al.* 2000), and FISH analysis with rDNA suggested that structural chromosome rearrangements have occurred (Kikuchi *et al.* 2008). Such sequence homology and structural differences in the chromosomes of these species are likely the cause of suppressed chromosome pairing. Ujihara *et al.* (1990) produced tetraploid interspecific hybrids between tetraploid *F. esculentum* and tetraploid *F. cymosum*. The tetraploid hybrids showed the same rate of bivalent chromosome formation and pollen production as the 4x parents, and they produced fertile BC1F1 plants. However, later generations are anticipated to be less fertile because of chromosome mismatch. Therefore, it would be necessary to clarify the mechanism of chromosome pairing and segregation, or devise crossing strategies, such as the production of amphidiploids by chromosome doubling, the use of doubled line as ‘Yokota-zairai’ that shows high probability of producing interspecific hybrids.

On the other hand, interspecific plants themselves which we developed seems to have value to investigate some traits which show difference between *F. esculentum* and *F. cymosum*, such as rutin contents. (The rutin contents of *F. cymosum* is much higher than that of *F. esculentum*.) In addition, interspecific plants would be good material for studying evolution and the evolution of cultivation, based on the perspective of chromosome reconstructions. Interspecific hybrid plants are currently maintained in culture. Recent breeding technologies such as transformation, TILLING and genome editing technology may be able to restore the fertility in near future and enable us to use the interspecific hybrids for breeding of buckwheat.

In conclusion, we developed and characterized interspecific diploid hybrids produced from a cross between *F. esculentum* and a related perennial species (*F. cymosum*). Furthermore, we identified the cause of sterility of the diploid hybrids, which were likely due to abnormal chromosome pairing and segregation during meiosis. Therefore, it would be difficult to obtain progeny from the diploid hybrids themselves; however, the production of amphidiploids by chromosome doubling could provide further breeding opportunities.

## Author Contribution Statement

MS, SK, YY, and KM conceived and designed the experiments. MS performed embryo rescue. YY and KM developed the DNA markers and checked the hybridity. MN and SK performed GISH analysis. MS, SK, YY, and KM wrote the manuscript, and all the authors read and approved the final manuscript.

## Supplementary Material

Supplemental Figure

Supplemental Table

## Figures and Tables

**Table 1.** Efficiency of the production of interspecific hybrids between *Fagopyrum esculentum* and *F. cymosum* using embryo rescue

Cross combination*	No. of ovules		Germination ratio (%)	No. of regenerated plants
	Cultured	Germinated		
Izumo-zairai × *F. cymosum*	68	0	0	0
Izumonomai × *F. cymosum*	30	1	3	0
Hitatiakisoba × *F. cymosum*	587	9	2	0
Matsue-zairai × *F. cymosum*	501	13	3	3
Yokota-zairai × *F. cymosum*	408	97	29	46

* Each mating combination was conducted for 1 or 2 years, and embryo development was observed. Individuals in which embryos emerged after breaking through the ovules were counted as germinating, and individuals in which the germinated embryos developed normally were counted as regenerated.
