# Supplementary material for: Development and chromosomal characterization of interspecific hybrids between common buckwheat (Fagopyrum esculentum) and a related perennial species (F. cymosum)
Source: Breed Sci. 2023 May 17;73(2):230–6. doi: 10.1270/jsbbs.22063 (PMC10316306; doi:10.1270/jsbbs.22063)
Supplement: Supplementary file 1 — Supplemental Figure [file 73_230_s1.pdf]

Sugiyama et al./Breeding Science
doi: 10.1270/jsbbs.22063

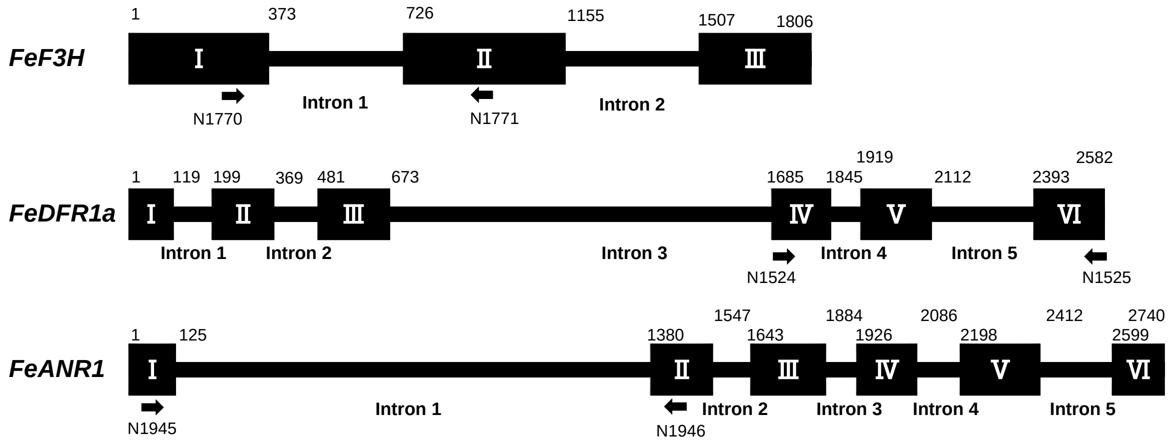

Supplemental Fig. 1. Structure of genes of *FeF3H*, *FeDFR1a* and *FeANR* based on genome databases.

*Boxes and lines indicate coding regions and introns, respectively. Roman numerals inside the boxes represent the number of the exons. Numbers above the boxes indicate the initial and final positions of the exons. Arrows indicate primer positions.
