# Supplementary material for: Development and chromosomal characterization of interspecific hybrids between common buckwheat (Fagopyrum esculentum) and a related perennial species (F. cymosum)
Source: Breed Sci. 2023 May 17;73(2):230–6. doi: 10.1270/jsbbs.22063 (PMC10316306; doi:10.1270/jsbbs.22063)
Supplement: Supplementary file 2 — Supplemental Table [file 73_230_s2.pdf]

Sugiyama et al./Breeding Science
doi: 10.1270/jsbbs.22063

Supplemental Table 1. Primers used to detect polymorphisms between *F. esculentum* and *F. cymosum*

Locus	Genbank No.	Genome Seq. (BGDB)	Primer combination	
			Forward (Primer Name, Sequence)	Reverse (Primer Name, Sequence)
FeF3H	HM149789.1	Fes_sc0005297.1	**N1770-FeFcF3HF01,** 5’-GTTGATACTAAGCTTGTTTCCGAG-3’	**N1771-FeFcF3HR01,** 5’-AACTCCAAGAAGCTTGCAAGCTAG-3’
FeDFR1a	LC216398.1	-*	**N1524-FeFcDFRF03,** 5’-CATTAGCATTATCCCAACTCTCGT-3’	**N1525-FeFcDFRR01,** 5’-TCAATGGCCATTACCATTTACATG-3’
FeANR1	LC107621.1	Fes_sc0002933.1	**N1945-FeFcANRF02,** 5’-TATTGCTTGAGAAGGGCTATTCTGTCAAC-3’	**N1946-FeFcANR02,** 5’-CTCATCAGTTAGATCAGCACGAAAGAT-3’

*, The previously reported data was used (Katsu et al. 2017).
